# Self‐Harm and Suicidal Ideation in Pregnancy: The Impact of Education on Knowledge, Confidence and Documentation Related to Screening and Follow‐Up to Question Ten‐Positive on the Edinburgh Postnatal Depression Scale

**DOI:** 10.1111/ajo.70180

**Published:** 2026-09-03

**Authors:** Wee Ming Wong, Ching Mei Loo, Susan Roberts, Grace Branjerdporn

**Affiliations:** ^1^ Mental Health and Specialist Services Gold Coast Health Southport Australia; ^2^ Faculty of Health Sciences and Medicine Bond University, University Drive Robina Australia; ^3^ Mater Research Institute, the University of Queensland Brisbane Australia

**Keywords:** depression, perinatal, pregnancy, screening, self‐harm, suicide

## Abstract

**Background:**

Suicide in the peripartum is a leading cause of death in Australia. Appropriate screening and response to self‐harm and suicidality during antenatal appointments is key to reducing the risk of suicide.

**Aim:**

The study explores the knowledge, confidence and behavioural changes resulting from an educational intervention for midwives in assessing and making appropriate referrals based on self‐harm and suicide risks in antenatal women, as screened by question ten of the Edinburgh Postnatal Depression Scale (EPDS) eliciting thoughts of self‐harm in the previous 7 days.

**Materials and Methods:**

Educational workshops aimed at improving midwives' confidence and capabilities in conducting suicide risk assessments, developing safety plans and understanding referral pathways were completed. Changes to medical documentation in relation to risk assessment and follow‐up actions were reviewed pre‐ and post‐ the education sessions.

**Results:**

Midwives' (*N* = 115) self‐reported knowledge and confidence increased from pre‐ to post‐education. Pre‐intervention, 86 (5.87%) antenatal women answered question ten positively on the EPDS. Medical documentation revealed that more midwives were able to screen for the frequency and persistence of suicidal thoughts following the training.

**Conclusion:**

While education enhanced knowledge and confidence as well as resulted in improvements to some risk assessment practices, further systemic and organisational support may be necessary to achieve consistent and comprehensive risk assessments and adherence to recommended follow‐up actions in clinical practice. The study underscores the critical role of continuous training for midwives in self‐harm/suicide assessment, particularly given the significant maternal mortality associated with suicide.

## 1 Introduction

In Australia, approximately 20% of mothers experience perinatal depression annually, affecting over 60,000 women [1, 2, 3]. Depression can also affect the woman's ability to bond with her child, resulting in potential insecure attachment of the child and social–emotional difficulties [2]. According to the Australian Institute of Health and Welfare (AIHW), suicide is a leading cause of maternal deaths in Australia [1, 4, 5]. Midwives make up the majority of the maternity care workforce in Australia and play a crucial role in detecting self‐harm and suicidal ideation during routine antenatal screening. By equipping midwives with knowledge and confidence in assessing and responding to self‐harm and suicide, this, in turn, can reduce the risk of suicide [1, 2, 4].

The Australian Clinical Practice Guideline, developed by the Centre of Perinatal Excellence (ACPG‐COPE) [2] recommends that women are routinely screened for depression using the Edinburgh Postnatal Depression Scale (EPDS) as early as possible in pregnancy and at least once later in pregnancy, as well as 6 to 12 weeks after birth [2, 3, 6]. The EPDS is widely implemented across Australian maternity services, with a case study of Queensland maternity services finding that over 89% of mothers have an EPDS score recorded in public hospitals and midwifery‐led models of care, compared to fewer than 40% of mothers under private specialist obstetrician care [7], demonstrating that midwives are central to the delivery of EPDS. The ACPG‐COPE also supports the assessment, treatment and management of prevalent mental health disorders and severe mental illnesses during the perinatal period [2].

The EPDS is effective as a patient‐reported screening tool for depression, but further assessment is still required for formal diagnosis [8]. Question 10 (Q10) on the EPDS specifically screens for thoughts of self‐harm in the previous 7 days, with selection either of ‘hardly ever’, ‘sometimes’ or ‘yes, quite often’ is considered Q10 positive. Women who answer positively to Q10 on the EPDS require timely mental health assessment, in keeping with ACPG‐COPE 2017 [2].

Although midwives are not expected to manage suicidal ideation, midwives are required to be skilled in assessing and determining referral pathways after completing a risk assessment [4]. Studies have shown that midwives' confidence and attitudes can impact their approach to screening and their actions following a positive screening result [9]. Haynes [10] emphasised the critical role of midwives in the early screening of perinatal mental illness during initial booking appointments. However, a study examining two approaches to psychosocial assessment found that midwives generally felt confident in conducting EPDS screening overall, except for asking about domestic violence and self‐harm (Q10) [11]. Midwives are the first health professional responsible for immediate escalation, safety planning and referral in line with national consultation and referral guidelines [12]. Midwives report relatively low positive confidence towards suicide prevention [9]; this confidence gap may compromise the consistency of escalation and follow‐up practice. A national survey of 800 Australian midwives found that despite routine use of EPDS, further training is needed to build competency and manage the needs of women identified through screening [13]. These findings suggest that significant gaps in training may hinder midwives' ability to perform these assessments effectively. Education around suicide safety planning and accessing acute mental health care may be helpful in improving midwives' knowledge and confidence [4].

A systematic review found that midwives at various stages of training and experience commonly reported negative attitudes towards caring for parents experiencing suicidal ideation, postpartum psychosis and severe mental illness. While the midwives recognised this as a part of their role, they felt underprepared and identified a need for further training [14]. The review also found that all the studies that implemented training programmes reported improvements in midwives' self‐rated knowledge, skills, attitudes and confidence in screening, referring and supporting parents. While education has been shown to improve midwives' broader perinatal mental health knowledge and confidence, no studies examine the effectiveness of education in improving a midwife's ability to complete safety planning and make appropriate referrals following a positive self‐harm (Q10) result on EPDS.

The aim of this clinical audit was to assess the effectiveness of an educational intervention for midwives in improving completion rates of EPDS screening, risk assessment and immediate management (including safety planning, follow‐up and referral practices) of Q10 positive in EPDS (with/without score ≥ 13) for self‐harm in women, and to evaluate the impact of the intervention on midwives' confidence and knowledge, in alignment with the ACPG‐COPE 2017 at the time of the study [2].

## 2 Methods

### 2.1 Study Design

This clinical audit was conducted in three stages (see Supporting Information 1). The study employed a repeated‐measures, cohort design. This study received exemption from ethical clearance by the Gold Coast Hospital and Health Service Human Research Ethics Committee (LNR/2020/QGC/61870).

### 2.2 Setting and Participants

The tertiary‐level public hospital in metropolitan Australia serves a population of 690,000 and has approximately 5000 births, making it the second‐highest birthing hospital [15]. The midwives provide comprehensive antenatal, intrapartum and postnatal services and a Perinatal Mental Health Service is available providing psychiatric consultations.

#### 2.2.1 Intervention

Two‐hour face‐to‐face education sessions were part of a 5‐h annual Perinatal Mental Health Education Workshop organised by the health service. In the year of this project, the workshop was allocated to the research investigators. The workshop was available to midwives and other healthcare professionals; participants were predominantly midwives. The intervention was delivered across three workshops about self‐harm/suicide risk assessment and management that were provided to midwives within work time. Investigators prepared and delivered a PowerPoint presentation on EPDS and suicide risk assessment, with materials adapted from the local Suicide Prevention Pathway (SPP) team [16] and other perinatal risk assessment training [17]. The education sessions covered the rationale for the EPDS, when and how to administer and score the EPDS, the importance of suicide risk assessment, how to conduct a suicide risk assessment when Q10 is positive, developing a safety plan and managing the outcomes of the risk assessment. The session also detailed health service referral pathways and provided examples of suicide risk assessment and management documentation. The sessions utilised PowerPoint presentations, interactive discussions and role‐playing of three clinical scenarios surrounding suicide risk assessment, safety planning and follow‐up measures with varying levels of risk.

### 2.3 Measures

#### 2.3.1 Documentation Review Pre‐ and Post‐Intervention

An audit of medical records of those who presented to health service antenatal clinics between July and December 2019 during the pre‐intervention period, and January to March 2021 during the post‐intervention period was completed. An audit tool (see Supporting Information 2) was created to collect clinical data on encounters such as EPDS completion, reasons for non‐completion, Q10 positive, completeness of risk assessment if Q10 positive and whether appropriate actions were taken according to risk assessment.

Completeness of risk assessment was based on the following criteria in accordance with ACPG‐COPE 2017 [2]: If self‐harm/suicidal thoughts are present, how frequent and persistent are they; if a plan has been considered; and if she has access to means. All three criteria needed to be fulfilled to be considered a complete risk assessment. Content analysis by two independent reviewers was undertaken to categorise the information from completed risk assessments into three categories: ‘low’ (fleeting thoughts of self‐harm or suicide but no current plan or means), ‘medium’ (suicidal thoughts and intent but no current or immediate plan) and ‘high’ (continual/specific suicidal thoughts, intent, plan and means) risk groups to determine the minimum follow‐up measures in accordance with recommendations in ACPG‐COPE 2017 [2] and local health service Perinatal Mental Health referral pathway (see Supporting Information 3) [18]. The level of appropriateness of follow‐up measures were undertaken according to the risk assessment (see Supporting Information 4 for an example of coding) was categorised as *yes, partially completed* or *no*. Discrepancies in coding were discussed between the two investigators until a consensus was reached. If further mediation was needed, a senior researcher (supervising Perinatal Psychiatrist) was consulted to resolve any remaining discrepancies.

#### 2.3.2 Midwifery Evaluation of Educational Intervention

Midwives attending the training completed a pre‐ and post‐session survey assessing participant's confidence and knowledge in conducting a risk assessment for self‐harm/suicide and its subsequent management (see Supporting Information 5). Participation in the survey was voluntary and completion was taken as implied consent. The online questionnaire consisted of demographic questions, and items surrounding subjective confidence (Part A) and objective knowledge (Part B), taking approximately 15 min to complete. Part A consisted of four questions and began with phrases like ‘I am confident in’ or ‘I have a clear understanding of’ and rated on a 10‐point Likert‐scale. Part B consisted of eight questions and included seven ‘true/false’ questions and one multiple choice question. To ensure de‐identification and matching across time, participants created unique codes for their questionnaires.

### 2.4 Statistical Analysis

STATA was used for analysis, and a biostatistician was consulted throughout. The following categorical variables were assigned a value of 0 or 1: each variable for ‘Criteria for risk assessment’ (0 = not assessed, 1 = assessed), *Risk assessment* (0 = incomplete, 1 = completed), ‘Follow‐up measures taken in accordance with risk assessment’ (0 = No, 1 = Yes) and ‘Follow‐up measures when risk assessment is incomplete’ (0 = No, 1 = Yes). Descriptive statistics were used to compute demographic data. Chi‐Square tests and Fisher's exact tests were used to evaluate changes in documentation. Generalised Estimating Equations (GEE) was used to evaluate differences pre‐ and post‐education questionnaires.

## 3 Results

**FIGURE 1:**
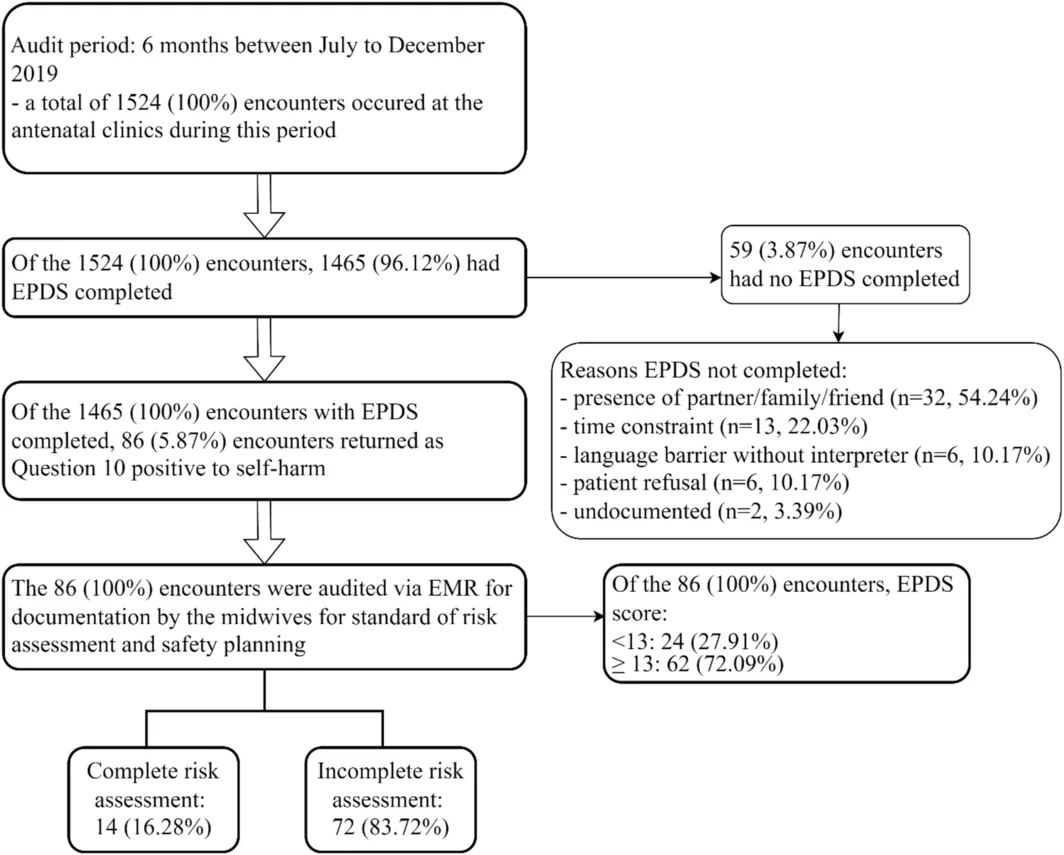
Pre‐intervention audit period including number of encounters, frequency and proportion of EPDS completion and reasons for non‐completion of EPDS.

As shown in Figure 1, during the pre‐intervention period, 1524 encounters were reviewed and 1465 (96.12%) completed the EPDS. There were 86 (5.87%) who answered positively on Q10 and 62 (72.09%) scored ≥ 13. Among those with positive Q10, 72 (83.72%) had incomplete risk assessments, while 14 (16.28%) had complete risk assessments of self‐harm/suicide ideation.

Reasons for non‐completion of the EPDS (3.87%, *n* = 59) in the pre‐intervention group were presence of partner/family/friend (54.24%), time constraints (22.03%), language barrier without interpreter (10.17%) and refusal from the patient (10.17%).

**FIGURE 2:**
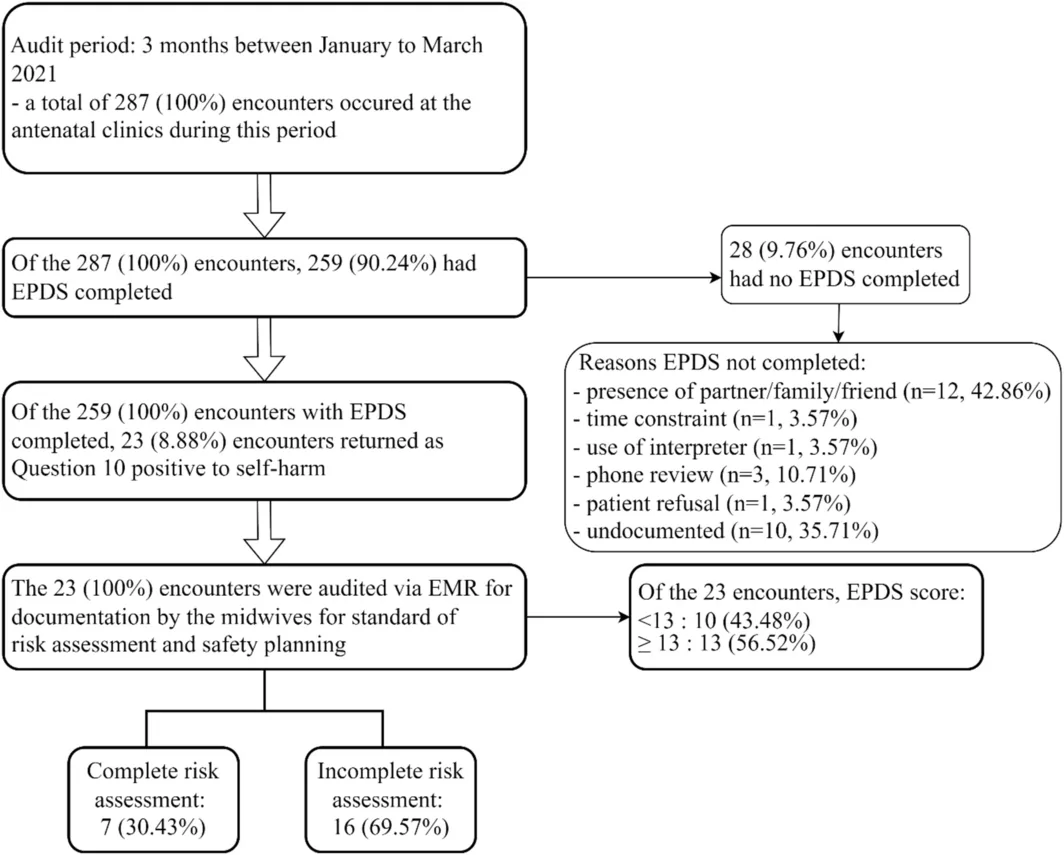
Post‐intervention audit period including number of encounters, frequency and proportion of EPDS completion and reasons for non‐completion of EPDS.

In Figure 2, the post‐intervention group had 287 encounters. Of these, 259 (90.24%) completed the EPDS and 23 (8.88%) answered positively to Q10, with 13 (56.52%) scoring ≥ 13. Of these 23 women, 16 (69.57%) had incomplete risk assessments, while seven (30.43%) had complete risk assessments.

Reasons for non‐completion of the EPDS (9.76%, *n* = 28) in the post‐intervention group were presence of partner/family/friend (42.86%), phone review (10.71%), refusal from the patient (3.57%), time constraint (3.57%) and use of interpreter (3.57%).

There was a significant increase in the rate of completion for the criterion assessing ‘if suicidal thoughts are present, how frequent and persistent they are’ from 25.6% pre‐intervention to 52.2% post‐intervention over the time period (see Table 1). Although the overall completion rate of risk assessments trended an increase from 16.3% to 30.4%, this was not statistically significant.

**TABLE 1:** Comparison of completion of documentation in accordance with risk assessment and the stratified level of appropriate follow‐up measures at pre‐ and post‐ intervention.

	Pre‐intervention proportion [95% CI]	Post‐intervention proportion [95% CI]	Difference (pre–post) [95% CI]	*p*
When EPDS Q10 positive to self‐harm				
Further risk assessment screening				
Suicidal thoughts—frequency, persistence	0.256 [0.168, 0.361]	0.522 [0.306, 0.732]	−0.266 [−0.490, −0.0419]	0.02
Plan	0.430 [0.324, 0.542]	0.304 [0.132, 0.529]	0.126 [−0.089, 0.341]	0.34
Means	0.186 [0.110, 0.285]	0.304 [0.132, 0.529]	−0.118 [−0.324, 0.087]	0.25
Completion of risk assessment				
Complete	0.163 [0.092, 0.258]	0.304 [0.132, 0.529]	−0.142 [−0.345, 0.062]	0.13
Incomplete	0.837 [0.742, 0.908]	0.696 [0.471, 0.868]	0.142 [−0.062, 0.345]	0.13
When risk assessment is complete				
Follow‐up measures taken in accordance with risk assessment variables for ‘low risk’ group^b^				
Discussed availability and treatment options	0.929 [0.661, 0.998]	0.714 [0.290, 0.963]	0.214 [−0.147, 0.575]	0.25
Arranged follow‐up/referral to PMH Service	0.857 [0.572, 0.982]	0.714 [0.290, 0.963]	0.143 [−0.239, 0.524]	0.57
Provide community resources	0.357 [0.128, 0.649]	0.571 [0.184, 0.901]	−0.214 [−0.659, 0.230]	0.40
Stratified level of appropriate follow‐up measures variable for ‘low risk’ group^b^				
Yes	0.357 [0.128, 0.649]	0.429 [0.099, 0.816]	−0.071 [−0.516, 0.373]	0.75
Partially completed	0.643 [0.351, 0.872]	0.429 [0.099, 0.816]	0.214 [−0.230, 0.659]	0.35
No	0.000 [0.000, 0.232]^a^	0.143 [0.004, 0.579]	−0.143 [−0.402, 0.116]	0.15
When risk assessment is incomplete, follow‐up measures provided				
Discussed availability and treatment options	0.764 [0.649, 0.856]	0.750 [0.476, 0.927]	0.014 [−0.220, 0.248]	0.91
Arranged follow‐up/referral to PMH Service	0.569 [0.447, 0.686]	0.438 [0.198, 0.701]	0.132 [−0.137, 0.401]	0.34
Provide community resources	0.403 [0.289, 0.525]	0.563 [0.299, 0.802]	−0.160 [−0.428, 0.108]	0.24
Contingency plan	0.028 [0.003, 0.097]	0.000 [0.000, 0.206]^a^	0.028 [−0.010, 0.066]	0.50
Develop a safety plan with the woman	0.028 [0.003, 0.097]	0.000 [0.000, 0.206]^a^	0.028 [−0.010, 0.066]	0.50

Abbreviations: Contingency plan, Have contingency plan in place for rapid re‐assessment if distress or symptoms escalate; PMH, Perinatal Mental Health; Provide community resources, Identify relevant community resources and provide contact details; Means, If she has access to means; Plan, If plan has been considered; suicidal thoughts, If suicidal thoughts are present, how frequent and persistent are they.

^a^ One‐sided 97.5% confidence interval.

^b^ ACPG‐COPE 2017 [1] risk assessment.

All women who answered positively on Q10 of the EPDS with completed risk assessment fell into the *‘low risk’* group in both the pre‐intervention (*N* = 14) and post‐intervention (*N* = 7) groups. According to ACPG‐COPE 2017 [2], ‘low risk’ women require follow‐up in the form of: (1) discuss availability of support and treatment options, (2) arrange follow‐up consultation and (3) identify relevant community resources and provide contact details. The rate of completion of ‘identifying community resources and providing contact details’ trended an increase from 35.7% pre‐intervention to 57.1% post‐intervention, although it was not statistically significant. There was also a non‐statistically significant increase in the rate of completion of all three follow‐up requirements from 35.7% pre‐intervention to 42.9% post‐intervention, signifying that the appropriate level of follow‐up measures was taken in accordance with risk. Seventy‐two women (83.72%) had incomplete risk assessments pre‐intervention, while 16 women (69.57%) had incomplete risk assessments post‐intervention. Risk stratification was not possible due to missing data.

### 3.1 Midwifery Evaluation of Educational Intervention

A total of 115 midwives attended the education sessions on self‐harm/suicide risk assessment. Midwives worked in a variety of settings including Birth Suite/Maternity Inpatient Unit (*n* = 45, 39.13%), the Newborn Care Unit (18, *n* = 15.65%) and antenatal care (*n* = 17, 14.78%). Most midwives (*n* = 53, 56.99%) had no prior training on the EPDS and self‐harm/suicide risk screening (see Supporting Information 6).

As detailed in Table 2, subjective confidence scores were statistically significantly higher post‐education than pre‐education for all items such as understanding in the importance of and confidence in screening for self‐harm and suicide during pregnancy, confidence in completing safety planning and understanding of referral pathways and available resources. Participants showed the highest improvement for Question 4 relating to safety planning. Overall, for knowledge scores, the mean number of correctly answered questions in Part B increased significantly from 6.538 [95% CI = 6.254, 6.823] pre‐session to 7.489 [95% CI = 7.337, 7.640] post‐session (*β* = 0.950, *p* < 0.001). In particular, the probability of a correct response statistically significantly increased for Question 9 (‘If a woman scores higher than zero on Q10 of the EPDS, immediate action is required’) and Question 10 (‘When completing the EPDS, you should ask the patient to circle the response that comes closest to how she has been feeling in the previous 30 days’).

**TABLE 2:** Summary statistics for Generalised Estimating Equations (GEE) based on pre‐and post‐education session confidence (Part A) and knowledge (Part B) questionnaire.

Question		Pre‐education mean (Part A)/Adjusted mean (Part B) [95% CI]	Post‐education mean (Part A)/Adjusted mean (Part B) [95% CI]	Coefficient (Part A)/Odds ratio (Part B) [95% CI]	*p*
Part A—Confidence Questions (10‐Point Likert Scale)					
2.	I am confident in screening for self‐harm and suicide in pregnancy	6.121 [5.650, 6.593]	8.048 [7.680, 8.416]	1.927 [1.574, 2.279]	< 0.001
3.	I have a clear understanding of the importance of screening for self‐harm and suicide in pregnancy	7.686 [7.235, 8.136]	9.196 [8.959, 9.433]	1.510 [1.099, 1.922]	< 0.001
4.	I am confident in completing a safety plan for someone who is having self‐harm thoughts or suicidal	4.910 [4.411, 5.408]	7.871 [7.495, 8.248]	2.962 [2.584, 3.339]	< 0.001
5.	I have a clear understanding of the referral pathways and resources available for women presenting with self‐harm and suicidal thoughts	5.278 [4.781, 5.775]	8.183 [7.868, 8.499]	2.905 [2.527, 3.283]	< 0.001
Part B—Correct answers^a^ to the knowledge questions when 0 = incorrect and 1 = correct (True/False response except Q8 which was multiple choice)					
7.	A woman answers ‘hardly ever’ to EPDS Q10. Since her answer does not suggest that she is currently suicidal, I do not have to explore further in terms of self‐harm/suicidal thoughts.	0.886 [0.820, 0.951]	0.816 [0.735, 0.897]	0.571 [0.279, 1.170]	0.126
8.	What is the EPDS?	0.854 [0.782, 0.926]	0.909 [0.849, 0.968]	1.702 [0.822, 3.524]	0.152
9.	If a woman scores higher than zero on Q10 of the EPDS, immediate action is required.	0.618 [0.519, 0.717]	0.897 [0.833, 0.960]	5.384 [2.522, 11.491]	< 0.001
10.	When completing the EPDS, you should ask the patient to circle the response that comes closest to how she has been feeling in the previous 30 days.	0.534 [0.433, 0.636]	0.967 [0.929, 1.005]	25.152 [7.719, 81.949]	< 0.001
11.	If a woman scores higher than zero on Q10 of the EPDS and will be seeing the Social Worker after her midwifery review, there is no need for further exploration of risk as she will be seen by the Social Worker directly after.	0.914 [0.857, 0.972]	0.977 [0.946, 1.009]	4.021 [0.805, 20.081]	0.090
12.	If a woman scores higher than zero on Q10 of the EPDS, I will need to explore risk of suicide as well.	0.925 [0.871, 0.979]	0.977 [0.946, 1.009]	3.482 [0.682, 17.773]	0.134
13.	Further actions are required if a woman scores higher than zero on Q10 of the EPDS if this is a repeated/follow‐up EPDS.	0.914 [0.857, 0.971]	0.977 [0.946, 1.009]	4.050 [0.810, 20.249]	0.088
14.	There is no further action if a woman scores higher than zero on Q10 of the EPDS and states that she feels safe returning home.	0.901 [0.841, 0.962]	0.966 [0.928, 1.004]	3.156 [0.928, 10.718]	0.065

*Note:* For Q1, participants created an ID to match surveys across pre‐ and post‐ timepoints. Adjusted means represent the probability of a correct response for the respective question.

^a^ See Supporting Information 5 Part B for the multiple choice selection and correct answer.

## 4 Discussion

Findings suggest that the education session may have contributed to improvements in midwives' confidence and knowledge, and this coincided with an increase in screening more frequently about the nature of suicidal thoughts. Similar studies have shown that educating staff can improve attitudes and confidence in suicide prevention [9, 19, 20, 21]. Using adult learning principles, midwives were encouraged to share their experiences in dealing with self‐harm/suicide, supported with problem‐solving and role‐playing scenarios [22]. The post‐education questionnaire (Part A) results indicated an improvement in their subjective confidence and knowledge regarding the topic, suggesting that the education was effective in improving self‐rated confidence and knowledge among attendees, though this cannot be generalised to the wider midwifery workforce given the limited training reach.

With women with completed risk assessments being categorised into the ‘low risk’ group, a common theme resulting in incomplete risk assessments was not addressing the ‘access to means’ criterion. The statewide guideline for suicide prevention practice reinforces the importance of assessing access to means in suicidal patients, as this has one of the greatest impacts on suicide reduction [16, 23].

Generally, midwives were comfortable discussing the availability of support and treatment options, arranging follow‐up consultations or referrals to Perinatal Mental Health, and identifying relevant community resources and providing contact details. However, they had difficulties making contingency plans for rapid re‐assessment if distress or symptoms escalated and developing a safety plan with the woman. Plausible reasons for this may include a lack of time as well as the midwives having limited confidence, knowledge and skills to complete this. Midwives are expected to be skilled in initial risk assessment, safety planning and determining appropriate referral pathways. This highlights the importance of education focusing on risk assessment and management for self‐harm/suicide for midwives, although midwives are not expected to manage suicidal ideation themselves; they are required to perform risk assessment and determine appropriate referral pathways. Without adequate training women at risk may not be identified or connected with the appropriate specialist care, potentially delaying access to mental health support, as well as addressing organisational barriers that inhibit these key actions [9, 24, 25, 26].

The rate of completion of EPDS, the pre‐ and post‐intervention audits indicated a high rate of completion (pre‐intervention = 96.12%, post‐intervention = 90.24%), similar to other Australian studies [27]. Among the reasons for non‐completion of EPDS were ‘language barriers’ and ‘use of interpreters’, cited in both pre‐ and post‐intervention audit periods. This could be mitigated by using translated EPDS, which have been shown to have good reliability and validity [28]. With the introduction of digital screening [29] which is available in 25 different languages, this may assist to mitigate language barriers.

The results underscore the need for increased training for midwives for self‐harm/suicide assessments. The EPDS specifically screens for self‐harm in Q10, making risk assessment and management crucial [6]. Suicide is a challenging topic for mental health workers, let alone midwives who are not considered specialist clinicians in this area. Organisational and systemic barriers such as limited time to extract large amounts of information from women at their booking appointment could be barriers to completing risk assessments and immediate management. Suicide prevention is a collective responsibility, and it is critical midwives are equipped with the necessary knowledge to address it effectively [4].

Limitations of the study were that the training reached only 33.82% of the total midwife cohort; documentation of midwives who had not received the educational intervention may have been included in the re‐auditing cycle. Further research examining the effects of online eLearning modules is warranted to increase uptake of training. As the data collection occurred during COVID‐19, this may have affected results given the psychological impacts on health professionals, physical distancing restrictions for staff education and change to phone reviews. Further research exploring the use of digital administration of EPDS may be useful. Finally, undocumented advice or follow‐up measures provided by midwives was not captured.

In conclusion, findings suggest that the education session improved midwives' confidence and knowledge in self‐harm/suicide risk assessment and appropriate follow‐up among those who attended. In practice, behavioural changes related to assessing suicidal thoughts more comprehensively were also observed. As suicide is a leading cause of maternal mortality, it is crucial to train midwives in risk assessment to ensure early identification and intervention for women at risk. Training equips midwives with the skills necessary to recognise warning signs of self‐harm and suicidal ideation, conduct safety plans and enable them to provide timely and appropriate referrals. By enhancing midwives' confidence and competence in these areas, this improves maternal mental health outcomes and reduces the incidence of maternal suicide.

## Funding

The authors have nothing to report.

## Conflicts of Interest

The authors declare no conflicts of interest.

## Supporting information


**Data S1:** ajo70180‐sup‐0001‐Supinfo.docx.

## Data Availability

The data that support the findings of this study are available from the corresponding author upon reasonable request.
