# Supplementary material for: Self‐Harm and Suicidal Ideation in Pregnancy: The Impact of Education on Knowledge, Confidence and Documentation Related to Screening and Follow‐Up to Question Ten‐Positive on the Edinburgh Postnatal Depression Scale
Source: Aust N Z J Obstet Gynaecol. 2026 Sep 3;66(5):e70180. doi: 10.1111/ajo.70180 (PMC13542300; doi:10.1111/ajo.70180)
Supplement: Supplementary file 1 [file AJO-66-0-s001.docx]

# **Supplementary Material 1**

**Figure.** *Three stages of the clinical audit including pre-, post- and intervention stage*

STAGE 1: PRE-INTERVENTION Chart audit via electronic medical records (EMR) for midwives' documentation of self-harm/suicide risk assessment and follow-up measures provided. This audit period was between July to December 2019 (six months).

STAGE 2: INTERVENTION Education sessions were provided to midwives focusing on conducting suicide risk assessment and follow-up measures to be provided, including safety planning. A pre- and post-session Confidence and Knowledge Questionnaire was conducted during the sessions.

STAGE 3: POST-INTERVENTION Re-audit one month post-intervention via electronic medical records (EMR) for midwives' documentation of self-harm/suicide risk assessment and follow-up measures provided. This audit period was between January to March 2021 (three months).

**Supplementary Material 2**

*Data collection tool used to assess EPDS completion and score, risk assessment if Q10 positive, and actions undertaken.*

1. Was the Edinburgh Postnatal Depression Scale completed?
   a. Yes
   b. No
2. What was the total score of the Edinburgh Postnatal Depression Scale?
   a. < 13
   b. ≥ 13
3. What was the answer for Question 10 (“The thought of harming myself has occurred to me in the past 7 days”) in Edinburgh Postnatal Depression Scale?
   a. Yes, quite often*
   b. Sometimes*
   c. Hardly ever*
   d. Never

* is considered positive to Q10

4. Was the level of risk assessed and documented if Q10 in Edinburgh Postnatal Depression Scale was positive?
   a. Yes
   b. No
5. If level of risk was assessed, what was the level of risk documented?
   a. Low
   b. Medium
   c. High
6. Were appropriate actions taken to manage the individual level of risks assessed if Q10 was positive?
   a. Yes
   b. Partially completed
   c. No

**Supplementary Material 3**

**Figure 2.** *Risk assessment criteria and subsequent follow-up measures in accordance with risk assessed*

CRITERIA FOR RISK ASSESSMENT

- If self-harm/suicidal thoughts are present, how frequent and persistent are they - If plan has been considered - If she has access to means

RISK STRATIFICATION IF OVERALL RISK ASSESSMENT COMPLETED

Low (fleeting thoughts of self-harm or suicide but no current plan or means)

Medium (suicidal thoughts and intent but no current or immediate plan)

High (continual/specific suicidal thoughts, intent, plan, and means)

FOLLOW-UP MEASURES IN ACCORDANCE WITH RISK AS PER ACPG-COPE 2017(2) AND LOCAL HEALTH SERVICE PERINATAL MENTAL HEALTH SERVICE REFERRAL PATHWAY(10)

LOW - Discuss availability of support and treatment options - Arrange follow-up consultation/referral to PMH+ - Identify relevant community resources and provide contact details

MEDIUM - Discuss availability of support and treatment options - Organise re-assessment within one week/referral to PMH+ - Have contingency plan in place for rapid re-assessment if distress or symptoms escalate - Develop a safety plan with the woman

HIGH - Ensure that the woman is in an appropriately safe and secure environment - Organise re-assessment within 24 hours and monitoring for this period/refer to Acute Care Team - Follow-up outcome of assessment - Monitor risk to infant

LEVEL OF APPROPRIATE FOLLOW-UP MEASURES

YES (all requirements for ‘follow-up measures taken in accordance with risk’ were completed)

PARTIALLY COMPLETED (some but not all requirements for ‘follow-up measures taken in accordance with risk’ were completed)

NO (all requirements for ‘follow-up measures taken in accordance with risk’ were incomplete)

*^α^ This flowchart is modelled after ACPG-COPE 2017^(^**^1^**^)^ and local health service Perinatal Mental Health Service Referral Pathway^(^**^2^**^)^*

*^+^Indicates Perinatal Mental Health*

References

1.	Austin M-P HN, and Expert Working Group. Mental Health Care in the Perinatal Period: Australian Clinical Practice Guideline 2017. Available from: chrome-extension://efaidnbmnnnibpcajpcglclefindmkaj/<https://cope.org.au/wp-content/uploads/2017/10/Final-COPE-Perinatal-Mental-Health-Guideline.pdf>.

2.	Service GCHaH. Perinatal Mental Health Service Referral Pathway – Community and Inpatient Referrals 2021.

# **Supplementary Material 4**

**Examples of Documentation of Midwives’ Risk Assessment if Q10 Positive and Relevant Coding**

| **Examples of documentation of midwives' risk assessment if Q10 positive** | **Total number of criteria completed for risk assessment based on documentation** | **Type of criteria completed based on risk assessment and documentation** |
| --- | --- | --- |
| Emotionally tired, EDPS 19 and positive to question 10 feels that she feels that things will be better if she just blends away. Does not feel like this today. | 0/3 | - None |
| EDS 23 - social worker notified. | 0/3 | - None |
| Reports has always struggled with thoughts of self-harm for 6 years but no intent to follow through. | 1/3 | - If self-harm/suicidal thoughts are present, how frequent and persistent are they |
| Describes thoughts of harming herself multiple times a week and states has decreased in severity in this pregnancy.  Protective factors discussed - states would never harm herself because of her boys + unborn baby. | 1/3 | - If self-harm/suicidal thoughts are present, how frequent and persistent are they |
| States hx of self-harm, struggling with thoughts around this. Has no immediate plans to self-harm. | 1/3 | - If plan has been considered |
| Reports having thoughts about 'not being here' once every few weeks but does not have a plan of how she would do it, nor does she intend to act on the thoughts. Reports when she has this thought she is able to stop it and think of something else. | 2/3 | - If self-harm/suicidal thoughts are present, how frequent and persistent are they - If plan has been considered. |
| Has ideas surrounding driving off the mountain on her way home, thinks of this often as it would be easier.  Asked to present to ED tonight, declines as has no intention of actually following through with it as has a 15 month old and is pregnant.  Does not feel like there is immediate concern and optimistic now she is seeking help. | 2/3 | - If self-harm/suicidal thoughts are present, how frequent and persistent are they - If plan has been considered. |
| Disclosed a hx of depression and borderline personality disorder. Extensive discussion around current emotional state- reports that she is emotionally well and that her answers to the EPDS reflect her natural/normal emotional state and that she is not concerned. Also disclosed a long hx of self-harming- cutting and nil suicidal ideation. Reported that she has good social and family support and that she is safe to go home with nil intention of harming herself however reports fleeting thoughts of cutting occurs to her occasionally. Reports the last time she self-harmed was over 6 months ago. | 3/3 | - If self-harm/suicidal thoughts are present, how frequent and persistent are they - If plan has been considered. - If she has access to means. |
| She says she has a long history of self-harm (superficial scars on arms and abdomen noted). She says she still has thoughts of harming herself but that this does not include suicide, and that she hasn't cut herself since being pregnant. | 3/3 | - If self-harm/suicidal thoughts are present, how frequent and persistent are they - If plan has been considered. - If she has access to means. |

**Examples of Midwives’ Documentation of Follow-up Measures and Relevant Coding**

| **Examples of midwives' documentation of follow-up measures** | **Type of management that was considered completed based on assessment of documentation** |
| --- | --- |
| Safe start referral | - Arrange follow-up consultation/referral to PMH. |
| Discussed ACAT numbers which she already has. | - None |
| Have given the Acute Care team brochure and encouraged her to contact them for assistance.  Have also given her the Pregnancy and Family Support Association Brochure.  Safe Start attended today with consent. | - Discussed availability and treatment options. - Arrange follow-up consultation/referral to PMH. - Identify relevant community resources and provide contact details. |
| Offered referral to Social work but declined. Given ACAT number. | - Discussed availability and treatment options. |
| Referred to midwife navigator and PMH. | - Discussed availability and treatment options. - Arrange follow-up consultation/referral to PMH. |
| Safety numbers and Acute care Team numbers given. Encouraged to speak up to us or her GP if she changes her mind and would like support.  Placed a Safe Start referral without consent. | - Discussed availability and treatment options. - Arrange follow-up consultation/referral to PMH. - Identify relevant community resources and provide contact details. |
| Safe start referral with consent. Not keen to go back to YHES house as she does not like big groups. Encouraged her to engage again and link in with their social worker. Also encouraged Benevolent society in Nerang for checks and engage with health worker for other child. Offered but declined meeting with social worker today.  Offered information on DV connect but feels that her ex-partner is not a threat at this stage and isn't concerned.  Offered AODS referral but declined as she stated she quit a week ago. | - Discussed availability and treatment options. - Arrange follow-up consultation/referral to PMH. - Identify relevant community resources and provide contact details. |
| Protective factors discussed including baby, partner, family and future career. Safety plan and ACT number discussed and given as well as reassured welcome to call MNGP if she has acute concerns about mental health.  Social work attended today's appointment for joint review. PMH referrral to be attended with consent. Encouraged to seek MH plan to see psychologist and other free options/resources discussed.  Anxiety management - breathing techniques/tracking current anxiety/panic attacks.  Strengths based counselling and problem solving  Education and Information Provision - relationships in pregnancy and risks post birth within DFV framework/  Psycho education - perinatal anxiety/depression, relationships in pregnancy, range of treatments re same.  Sent email information/resources following visit today - Acute Care Team, Psychology in community, PFS, Anxiety Management worksheets fact sheets, GCYS ASP accommodation program, YHES House, Keeping Well Booklet, Benevolent Society (has DV purple book from MW previously as well as DVPC supports) | - Discussed availability and treatment options. - Arrange follow-up consultation/referral to PMH. - Identify relevant community resources and provide contact details. - Have contingency plan in place for rapid re-assessment if distress or symptoms escalate. - Develop a safety plan with the woman. |
| Referral to Social Worker.  Referral to Perinatal Mental Health  Referral to Child Health referral completed  Referral to Benevolent society for further community support completed.  Partner aware to call for assistance if any worries. | - Discussed availability and treatment options. - Arrange follow-up consultation/referral to PMH. |
| Referred to SW.  Reinforcing access to services such as 13HEALTH, PANDA, Raising children’s network, mybody mybaby websites. | - Discussed availability and treatment options. - Identify relevant community resources and provide contact details. |
| Referred to SafeStart.  Encouraged to attend her counsellor and given resources such as PANDA, BeyondBlue and headspace.  Gave Community safety card and advised she could call ACT at anytime or present to ED if required. Offered to call ACT today but declined. | - Discussed availability and treatment options. - Arrange follow-up consultation/referral to PMH. - Identify relevant community resources and provide contact details. |

# **Supplementary Material 5**

**Pre- and Post-Education Session Confidence and Knowledge Questionnaire**

**Part A**

For each of the question below, please select the response that best characterises how you feel about the statement:

10 = Very high confidence

1 = No confidence at all

2. I am confident in screening for self-harm and suicide in pregnancy.

| 1 | 2 | 3 | 4 | 5 | 6 | 7 | 8 | 9 | 10 |
| --- | --- | --- | --- | --- | --- | --- | --- | --- | --- |

3. I have a clear understanding of the importance of screening for self-harm and suicide in pregnancy.

| 1 | 2 | 3 | 4 | 5 | 6 | 7 | 8 | 9 | 10 |
| --- | --- | --- | --- | --- | --- | --- | --- | --- | --- |

4. I am confident in completing a safety plan for someone who is having self-harm thoughts or suicidal.

| 1 | 2 | 3 | 4 | 5 | 6 | 7 | 8 | 9 | 10 |
| --- | --- | --- | --- | --- | --- | --- | --- | --- | --- |

5. I have a clear understanding of the referral pathways and resources available for women presenting with self-harm and suicidal thoughts.

| 1 | 2 | 3 | 4 | 5 | 6 | 7 | 8 | 9 | 10 |
| --- | --- | --- | --- | --- | --- | --- | --- | --- | --- |

6. How many hours of training regarding EPDS and self-harm/suicide risk screening have you attended before this?

Enter your answer:

**Part B**

Please select the correct answer for the following questions:

7. A woman answers "hardly ever" to EPDS Q10. Since her answer does not suggest that she is currently suicidal, I do not have to explore further in terms of self-harm/suicidal thoughts.

True

False*

8. What is the EPDS?

a. A 20-question depression screening tool used in the postpartum period only

b. A 10-question tool that screens for perinatal depression and is only available in English

c. A 10-question tool that screens for perinatal depression*

d. A 10-question depression screening tool used in the third trimester only

9. If a woman scores higher than zero on question 10 of the EPDS, immediate action is required.

True*

False

10. When completing the EPDS, you should ask the patient to circle the response that comes closest to how she has been feeling in the previous 30 days.

True

False*

11. If a woman scores higher than zero on question 10 of the EPDS and will be seeing the Social Worker after her midwifery review, there is no need for further exploration of risk as she will be seen by the Social Worker directly after.

True

False*

12. If a woman scores higher than zero on question 10 of the EPDS, I will need to explore risk of suicide as well.

True*

False

13. Further actions are required if a woman scores higher than zero on question 10 of the EPDS if this is a repeated/follow-up EPDS.

True*

False

14. There is no need for further actions if a woman scores higher than zero on question 10 of the EPDS and states that she feels safe returning home.

True

False*

Note: * Indicates the correct answer

# **Supplementary Material 6**

*Descriptive statistics of participants attending education sessions on self-harm/suicide risk assessment and management (N=115).*

|  |  | n | % | Mean | Median | SD | Min | Max |
| --- | --- | --- | --- | --- | --- | --- | --- | --- |
| Clinical role | | 115 | 100.00 |  |  |  |  |  |
|  | Birth suite/Maternity Inpatient Unit | 45 | 39.13 |  |  |  |  |  |
|  | Newborn care unit (RN’s) | 18 | 15.65 |  |  |  |  |  |
|  | Antenatal only | 17 | 14.78 |  |  |  |  |  |
|  | Maternity education team | 9 | 7.83 |  |  |  |  |  |
|  | Maternity home visit only | 8 | 6.96 |  |  |  |  |  |
|  | Midwifery Group Practice | 5 | 4.35 |  |  |  |  |  |
|  | Others* | 13 | 11.30 |  |  |  |  |  |
| Years in clinical practice | | 88 | 76.52 | 13.47 | 8.50 | 12.71 | 0.00 | 39.00 |
|  | <2 | 12 | 13.64 |  |  |  |  |  |
|  | 2–4 | 5 | 5.68 |  |  |  |  |  |
|  | 5–9 | 13 | 14.77 |  |  |  |  |  |
|  | >9 | 43 | 48.86 |  |  |  |  |  |
|  | Did not answer | 6 | 6.81 |  |  |  |  |  |
|  | Not midwife | 9 | 10.23 |  |  |  |  |  |
| Hours of training prior^+^ | | 93 | 80.87 | 2.53 | 0.00 | 5.58 | 0 | 30 |
|  | 0 | 53 | 56.99 |  |  |  |  |  |
|  | 1–2 | 20 | 21.51 |  |  |  |  |  |
|  | 3–5 | 5 | 5.38 |  |  |  |  |  |
|  | 6–9 | 8 | 8.60 |  |  |  |  |  |
|  | 10–12 | 3 | 3.23 |  |  |  |  |  |
|  | >12 | 4 | 4.30 |  |  |  |  |  |

^α^Descriptive statistics for total number of participants across three workshops
*Including casual pool staff, Alcohol and Other Drug Services staff, Antenatal midwives from other health services, and medical students
^+^Any prior hours of training regarding EPDS and self-harm/suicide risk screening
